# The Development of Delta: Using Agile to Develop a Decision Aid for Pediatric Oncology Clinical Trial Enrollment

**DOI:** 10.2196/resprot.9258

**Published:** 2018-05-04

**Authors:** Eden G Robertson, Claire E Wakefield, Richard J Cohn, Tracey O'Brien, David S Ziegler, Joanna E Fardell

**Affiliations:** ^1^ Behavioural Sciences Unit Kids Cancer Centre Sydney Children's Hospital Randwick Australia; ^2^ Discipline of Paediatrics School of Women’s and Children’s Health University of New South Wales Kensington Australia; ^3^ Kids Cancer Centre Sydney Children's Hospital Randwick Australia

**Keywords:** decision support techniques, decision making, neoplasms, patients, child, patient portals

## Abstract

**Background:**

The internet is increasingly being used to disseminate health information. Given the complexity of pediatric oncology clinical trials, we developed Delta, a Web-based decision aid to support families deciding whether or not to enroll their child with cancer in a clinical trial.

**Objective:**

This paper details the Agile development process of Delta and user testing results of Delta.

**Methods:**

Development was iterative and involved 5 main stages: a requirements analysis, planning, design, development, and user testing. For user testing, we conducted 13 eye-tracking analyses and think-aloud interviews with health care professionals (n=6) and parents (n=7).

**Results:**

Results suggested that there was minimal rereading of content and a high level of engagement in content. However, there were some navigational problems. Participants reported high acceptability (12/13) and high usability of the website (8/13).

**Conclusions:**

Delta demonstrates the utility for the use of Agile in the development of a Web-based decision aid for health purposes. Our study provides a clear step-by-step guide to develop a Web-based psychosocial tool within the health setting.

## Introduction

### Decision Making Regarding Pediatric Oncology Clinical Trials

Many parents are faced with the decision of whether to enroll their child with cancer in a clinical trial. In Australia, approximately 80% of children with cancer enroll in a clinical trial. Despite the necessity of clinical trials to test new treatments with the aim to find more effective cancer treatments, patients and parents often find the decision difficult [1,2]. Families often find the rationale, design, and long-term implications of participating in trials difficult to understand [3]. Early phase clinical trials can be particularly confusing, with parents and young people overestimating the potential benefit of the trials (called therapeutic misconception) [4,5], making it difficult for them to weigh up the benefit and burden of enrolling while hoping for a cure. Deciding whether to enroll is also complex, given the time pressures to proceed to therapy, the large amount of information to comprehend, and the emotional timing of the decision, which is often at either diagnosis or relapse. Many families experience decisional anxiety and uncertainty and psychological distress associated with the decision whether to enroll their child in a clinical trial or not [1,6].

With clinical trial decisions, there may not necessarily be a *right or wrong* choice of treatment. In these scenarios, individual values and preferences of the family become crucial. Decision aids are evidence-based tools designed to assist clients to be involved in making specific and deliberated choices among health care options [7]. Decision aids support patients and caregivers to make informed decisions by helping them to balance their values with the benefits and disadvantages of their treatment options. The “International Patient Decision Aid Standards” (IPDAS) highlight the role of clear information of treatment options based on research evidence, opportunities to clarify and express values, structured guidance in deliberation and communication of choice, and developed using a systematic development process [8]. Although decision aids vary in formats, decision aids appear to improve knowledge, quality of informed consent, and decision satisfaction for adult patients making treatment or screening decisions (eg, prostate cancer screening, menopausal hormone therapy) [9]. An evaluation of a decision aid in adult cancer clinical trials (“Cancer Research Choices”) has also shown reduced decisional conflict and postdecision regret compared with standard of care, without impacting clinical trial enrollment rates [10]. Despite the fact that some decision aids exist in adult clinical trials, none are available in pediatric oncology.

### Agile Development Process

Agile development is an overarching term to describe a software development process. Agile focuses on collaborations between developers and stakeholders, flexible methodology, and the ability to respond quickly to change through multiple iterations [11,12]. Changes even in late development are encouraged. This approach aims to deliver a product that can quickly adapt to clients’ changing needs and also deliver a high-quality and high-value project within the constraints (ie, cost, schedule, and scope) [13].

The use of Agile approaches has an increasing worldwide use within software development [14,15] and research [16]. Use of Agile approaches has been reported to result in higher business performance, customer satisfaction, and product quality compared with more structured nonfluid approaches [17]. Agile projects were reported to be 28% more likely to succeed (defined by a project that is completed on time and budget and includes originally outlined specifications) [18]. Multiple case studies have acknowledged the challenges of implementing Agile development, such as highly intensive interactions with stakeholders [19], however many have reported an overall positive impact [20-23].

In recent years, there has been a rapid growth in the use of, and access to, the internet for health purposes [24,25]. In Australia, studies have reported over 28% of patients access Web-based health information [26], and in the United States, more than 43% do [27]. In Swedish adult cancer patients, approximately 77% of patients seek Web-based health information [28]. Accessing health information in oncology has been reported to improve oncologist-patient communication and connectedness, patient use of medical jargon, and scientific knowledge [29].

Given the movement toward a more patient-centered and collaborative decision making approach in the health care system [30], there is a rising need for innovative product development that is provided online and tailored to the patient and their family. The principles of Agile are an appropriate vehicle to guide the development of a Web-based patient decision aid, especially those with clear aims yet flexibility in design [11].

## Methods

### Using Agile to Develop Delta

We developed Delta, a Web-based decision aid to support families deciding whether or not to participate in a pediatric oncology clinical trial. The main of aim of Delta was to improve clinical trial knowledge and facilitate treatment discussions and shared decision making between families and health care professionals (HCPs). Families offered a clinical trial are able to log in to the Delta website, which links to the clinical trial they are considering. Delta incorporates general content about clinical trials (eg, what are clinical trials, how do clinical trials work), the specific clinical trial information sheet, and a values clarification exercise. This paper provides a detailed description of the development and early testing of Delta, acting as proof of concept for the use of Agile for the development of a Web-based decision aid.

**Figure 1:**
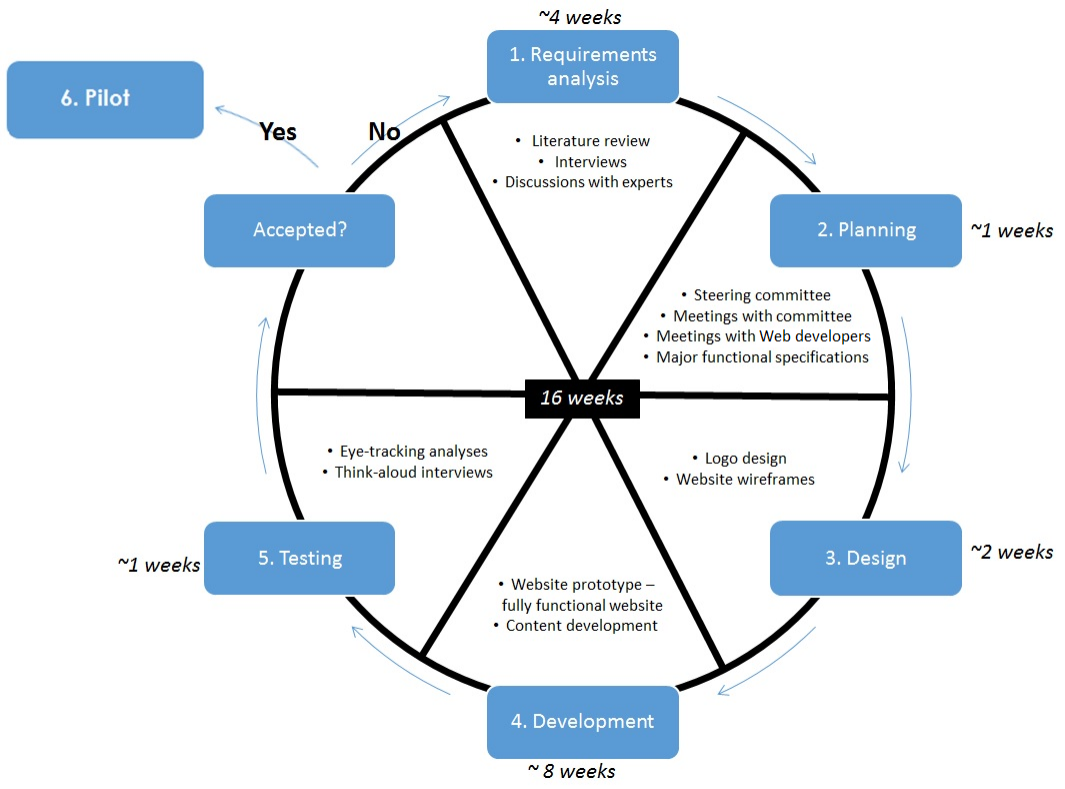
Delta development process. Estimated time is based on full days of work required, which occurred over a 12-month period.

The development of Delta was divided into the 5 main stages involved in Agile development and was both iterative (with one iteration referring to revisions made on one particular version of the website following feedback) and incremental (see Figure 1). We expected development to take 4 months from approval of the functional specification, with an additional 2 months for prepilot user testing. Due to delays in development and complexity of using Agile in research (see Discussion section), the actual development time of Delta was approximately 4 months of full-time work and 2 months of user testing, spread over 12 months. Our development was driven by guidelines for patient websites [31], the International Patient Decision Aid Standards (IPDAS) [7], and 12 Agile principles (see Multimedia Appendix 1).

The development of Delta was supported by a steering committee (16 members) to ensure development followed IPDAS [7]. The committee included psycho-oncology researchers (n=6), pediatric oncologists (n=5), a pediatric oncology clinical trial research manager (n=1), a clinical nurse consultant (n=1), parents (n=2), and a young person with cancer (n=1). All consumers signed a Terms of Reference Agreement specifying the purpose of the committee and their role. As Agile leans toward a leadership and collaboration approach, where a team leader acts as a facilitator, we also established a lead development team (3 members) from the steering committee to provide more regular feedback (ER, CW, and JF). The lead team met with each other face-to-face fortnightly, and all steering committee members met on an as-needed basis.

### Requirements Analysis

Requirements analysis was required to identify the needs and preferences of families in regard to information and functionality of the website. We identified needs and preferences of families by conducting a basic literature search, leading a systematic review on strategies to facilitate shared decision making in pediatric oncology clinical trials [32], and through discussions with the steering committee. Our team also conducted interviews with 25 Australian parents and 5 adolescents recently diagnosed with cancer to identify the needs and preferences of families when making treatment decisions. We continued the requirements analysis throughout the whole development process via discussions with the steering committee and literature searches.

### Planning

Planning was required to determine appropriate timelines for the completion of sprints (ie, smaller milestones to be completed and reviewed in short time frames), content and website functions, and design. We chose Web developers based on their experience, reputation from previous clients, corresponding values in development ideologies, cost of deliverables, and workable time frames. Planned sprints, between 2 and 4 weeks, were also agreed upon based on workable time frames of the lead team and Web developers. A lead team member (ER) developed the content framework and the ideal design and functionalities of the website, which was approved after 3 iterations by the other lead team members. ER met with Web developers face-to-face on 3 occasions before beginning development to discuss the website concept, functional specifications, and website requirements. Web developers then finalized the functional specification, which the lead team approved.

### Design

The design stage involved design of the Delta logo, color scheme, font, and graphics and website wireframes (ie, a visual display of the function and framework of the website). A graphic designer with a good working relationship with the lead team assisted with these tasks. The logo and color scheme went through 2 iterations before approval by the lead team and steering committee. ER developed wireframes following the initial planning with the Web developers. Wireframes went through 3 iterations with the lead team before beginning functional website protocol development. Web developers then developed the website prototype. We asked all steering committee members to provide feedback on the website prototype. We implemented suggested modifications based on consensus.

### Development

The development stage involved the development of content and the functional Delta website. We developed Delta to be highly scalable, both horizontally (ie, ability to add more resources, such as content) and vertically (ie, ability to increase the functionality or capacity, such as potential functionality as an app). Delta has a responsive Web design. It has been developed to be user-friendly across multiple platforms, including computers, tablets, and mobile phone, and has in place the capability to be scaled up to be available as an app. Although currently only in English, it also has the capability to be scaled out to be available in multiple languages. Delta can also be easily scaled out by allowing the addition of as many clinical trials as necessary. The method to add in additional clinical trials has been simplified to ensure Delta is easily maintainable. We also ensured Delta was developed with a content management system, which allowed content to be easily updated without the need for Web developers.

#### Content Development

Content went through approximately 8 rounds of iterations across 6 months, before being embedded within the website. We developed 2 versions of Delta (one version for parents and one for adolescents aged 12 years and older). Our systematic review [32] identified 3 key strategies to facilitate shared decision making in pediatric oncology: (1) quality information exchange, (2) clear communication, and (3) decision making support. We developed Delta to incorporate these 3 core features of shared decision making [32]. We developed Delta to cater for low health literacy populations. To facilitate information exchange, we presented information at Grade 8 readability for parent content [33] and Grade 5 readability for adolescent content. The reader is able to access less or more detailed information based on their preference for information amount. We provided an option grid for readers to easily compare key information between their options. We also acknowledged the role of gist memory (that is, recall of bottom-line meaning rather than detailed information) and therefore included summary boxes of information. To promote communication, we incorporated a question prompt list and suggested strategies for parents to communicate with HCPs and their child (if appropriate).

To provide more decisional support, we incorporated a values clarification exercise. A values clarification exercise allows participants to clarify and communicate the personal value of options, to ultimately make a better quality decision [34] that is most personally desirable [35], and results in less decisional regret [36]. The Delta values clarification exercise allows parents to rate their reasons to enroll or not to enroll in the clinical trial on a scale of personal importance. Delta is able to show whether the parent appears to be leaning toward enrolling in the trial or not. As per many patient decision aids [37], the algorithm for the outcome of the Delta values clarification exercise is basic, providing a summation of participant responses. Although items may be weighted differently for each participant, the purpose of the decision making exercise is not to provide a *correct answer* or a *recommendation*, but rather allow participants to weigh up their treatment options. Participants are instructed that this exercise will provide some indication as to the option they are more inclined to choose, but to discuss it further with their treating team.

Delta also provides the specific information sheet for the clinical trial in which the family was invited to participate. The website also includes a glossary, a text-to-speech function, and the ability to save and print notes from within the website. See Table 1 for the key elements of Delta.

#### Web Development

Web developers delivered progress in fortnightly or monthly sprints. The lead team provided feedback of the sprints, and Web developers made necessary modifications as they came up. For the final sprint, of the 16 steering committee members, 12 were available to provide feedback. Modifications suggested by the steering committee were discussed with the lead team and implemented based on consensus. We received multiple iterations of the website in early stages of development from steering committee members. During later stages of development, the lead team requested less feedback and thus had fewer iterations. Fewer iterations in later development were due to significant delays in development and the minimal value that later iterations provided (eg, minor wording changes). See Multimedia Appendix 2 for a video introduction to the Delta website.

**Table 1:** Key elements of Delta, categorized by function and health literacy needs.

Element	Strategy theme^a^	Health literacy^b^
Information about clinical trials (grade 8 readability and minimal medical jargon)	Information provision	Functional
Specific clinical trial information sheet	Information provision	Functional
Interactive glossary	Information provision	Functional
Text-to-speech functionality	Information provision	Functional
Question prompt list	Communication	Communicative
Personal notes page	Communication	Communicative
Communication strategies	Communication	Communicative
Values clarification exercise	Decision making support	Critical

^a^Strategy themes were identified via our systematic review [32].

^b^Health literacy categories are based on Nutbeam’s model of health literacy [38].

### User Testing

We conducted eye-tracking analyses to assess the usability of the Delta website. Eye-tracking analyses have frequently been used in website development [39,40]. The analysis used a computer with an embedded webcam to track each participant’s point of gaze. We conducted eye-tracking analyses to determine where participants’ gaze lingered, their length of gaze, and readability (ie, scanning, reading, or rereading of content). Data collected through eye-tracking analyses can be presented as either a gaze plot or a heat map. Gaze plots show the location, order, and time (known as the fixation duration) spent looking across certain aspects of the website. Heat maps are a visualization of the focus of visual attention across multiple participants.

We also conducted retrospective think-aloud interviews to establish participant satisfaction and acceptability of the website. Retrospective think-aloud interviews encourage participants to verbalize aloud their thought processes on replay of their task completion and are commonly used in website development [41,42]. The literature suggests 5 to 9 participants in think-aloud interviews, and eye-tracking analyses can detect 80% to 90% of usability problems on a website [43,44].

Individuals were eligible to participate if they were a parent of a child treated for cancer at Sydney Children’s Hospital. ER undertook user testing in a private office at the Sydney Children’s Hospital. Participants were all experienced at using computers and the internet. Participants were reimbursed with an Aus $20 gift voucher. The local ethics committee approved the user testing.

We instructed participants to browse the website for a maximum of 40 min. We asked participants to view the website as if they had had a consultation with their clinician regarding enrolling in a clinical trial and then provided with the Web-based decision aid. We emphasized to participants that the goal was not to assess their computer skills or their knowledge of clinical trials but rather to test the usability and acceptability of the website. If participants did not view the values clarification exercise of their own accord, ER directed the participant to complete it. Following the free browsing of the website, ER asked basic questions regarding usability, functionality, and acceptability of the website. We used a retrospective think-aloud protocol. This involved participants thinking aloud their thought process alongside a visual replay of their own eye-tracking data.

## Results

### User-Testing Results

A total of 17 families were identified as appropriate to contact by an oncologist or nurse (see Table 2 for participant demographics). Moreover, 7 parents opted into the study (all mothers). Reasons for nonparticipation included being too busy or living too far away to come to the hospital. Eye-tracking data are missing for one parent because of technical difficulties. A total of 6 HCPs in psycho-oncology (4/6 females) also participated in user testing.

Eye gaze was detectable, on average, 93% of the time. Validity of the gaze for both the left and right eyes was 0 on average, suggesting that the tracking quality was good. Participants left-clicked 142 times on average (range 86-237 clicks). Participants took 50.1 s to log in to the website (SD 31.0; range 25-133), 14.3 min on average to read the general content of decision aid (SD 9.5; range 2.1-33.6), and 2.5 min to complete the values clarification exercise (SD 0.9; range 1.1-4; see Table 3 for an overview of data).

Most participants completed the values clarification exercise (5/6 HCPs; 6/7 parents), with 5 participants (4 HCPs, 1 parent) accessing the exercise without being prompted. The reasons participants did not access the exercise was because they did not see the link to access the exercise (n=4 parents) or did not feel it was relevant to them (n=1 HCP). One HCP completed the exercise twice as they wanted to see if their responses changed after reading more information. The methods of accessing content were to use the side panel menu (n=3 HCPs, n=3 parents), the “next” button (n=1 HCPs, n=2 parents), a combination of both (n=2 HCPs), or the home button (n=1 parent).

**Table:** Summary of parent participant characteristics.

Parental role	Child age at diagnosis	Time since child’s diagnosis in months	Clinical trial^a^
Mother	12	12	No
Mother	7	12	Yes
Mother	6	11	Yes
Mother	0.5	54	Yes
Mother	7	33	Yes
Mother	5	58	Yes
Mother^b^	13	6	Yes
Average (SD)	7.2 (4.2)	26.6 (21.9)	N/A^c^

^a^Families who were enrolled in a clinical trial as part of their child’s cancer treatment.

^b^Eye-tracking data are missing for this participant because of technical issues.

^c^N/A: not applicable.

**Table 3:** Overview of time to complete tasks.

Tasks	Overall (n=12)		Parent (n=6)		Health care professionals (n=6)		*t* value^a^ (degrees of freedom)
	Average time (SD)	Range	Average time (SD)	Range	Average time (SD)	Range	
Log in time	50.1 s (31.0)	25-133 s	64.4 s (40.8)	27-133 s	38.2 s (14.9)	25-65 s	−1.475 (9)
Reading general content	14.3 min (9.5)	2.1-33.6 min	13.7 min (7.3)	5.3-24.0 min	14.8 min (12.0)	2.1-33.6 min	0.181 (10)
Completing the values clarification exercise	2.5 min (0.9)	1.1-4 min	2.8 min (0.7)	1.8-3.7 min	2.1 min (1.0)	1.1-4 min	0.424 (10)

^a^No comparisons were significant.

Most participants reported high acceptability (5/6 HCPs; 7/7 parents) and high usability of the website (4/6 HCPs; 4/7 parents):

It was nice and clear. It was simple. It had a good flow, not too many buttons to make you completely overwhelmed. Your emotions would be ridiculously high if you are looking at this, so you need to make it as simple as possible, so I like that there are three main parts to it

Parent of a 5-year-old boy with neuroblastoma

Most participants (5/6 HCPs; 6/7 parents) found the content easy to read and reported that it flowed logically. Some participants read the summary boxes before reading the main content (2/6 HCPs; 2/7 parents). These participants chose to read that section first as they felt it would be the most important information. Minor word changes were suggested by all participants (eg, change “me time” to “self-care”). None of the participants used the personal notes function, however several freely reported in the think-aloud interview that this function would be useful (n=1 HCP; n=3 parents). Moreover, 7 participants used the glossary (n=3 HCPs; n=4 parents), reporting the usefulness of the function. No participants used the speech-to-text function.

**Figure 2:**
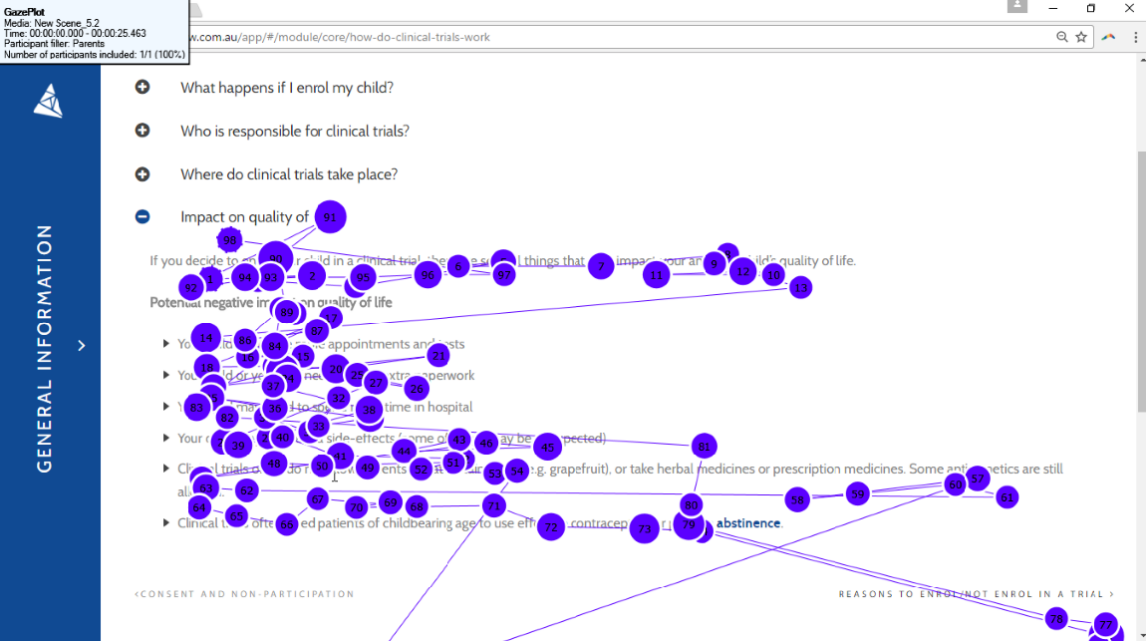
Gaze plot of one participant. Circles represent the gaze or fixation of the participant, with larger circles indicating longer fixations. Circles are numbered based on the order of fixation. The line between each fixation point represents the saccade or rapid movement between each fixation. This participant is reading lines from start to finish and appears to understand content as there are few long fixations. Only one participant was able to be used in this gaze plot as Delta is a scrolling website, and other participants did not stop their webpage in the exact same location.

**Figure 3:**
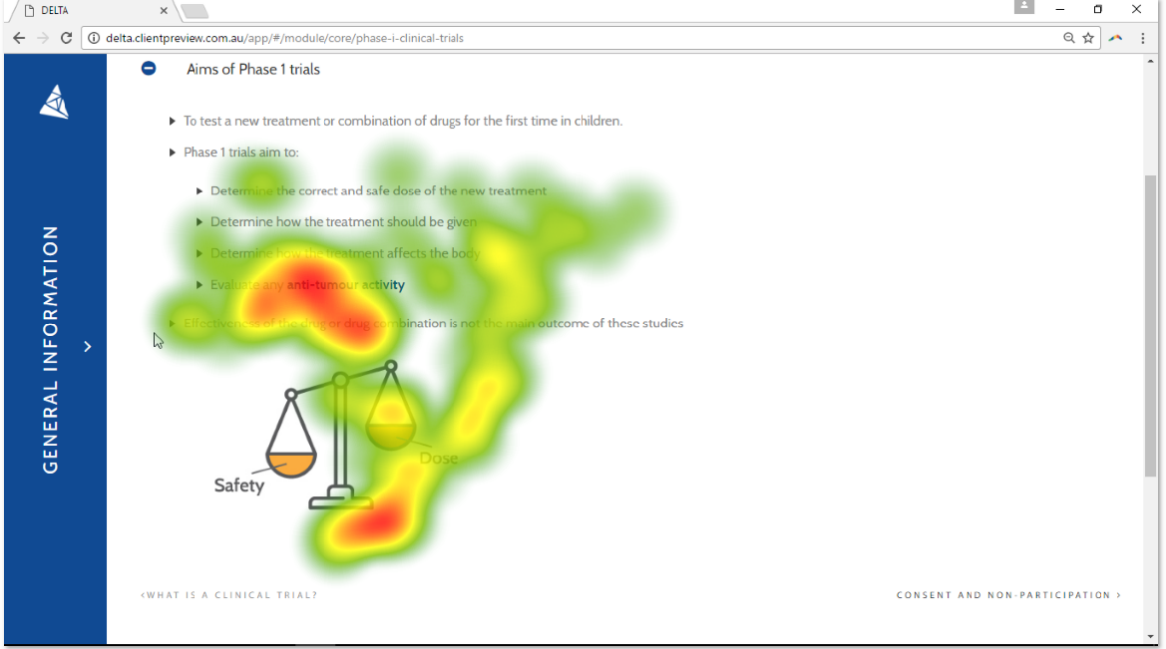
Heat map of one participant. Color represents the length of gaze, with deep red indicating longer fixations. Longer fixation on “antitumor activity” suggests higher levels of cognitive loading and potentially reduced comprehension.

There were few instances of rereading of content, with eye-tracking data suggesting that participants read lines to completion (see Figure 2). Most participants (5/6 HCPs; 6/6 parents; n=1 missing parent) skipped at least one page of content. Participants skipped content as they either did not want to read that information (1/5 HCPs; 1/6 parents) or did not realize they had skipped it (4/5 HCPs; 5/6 parents). Participants suggested that reducing overlap of images and phrases would improve usability. There were some particularly long fixations throughout the website, which is indicative of cognitive processing. Examples of long fixations include the terms “antitumor activity” and “dose escalation” (see Figure 3).

**Figure 4:**
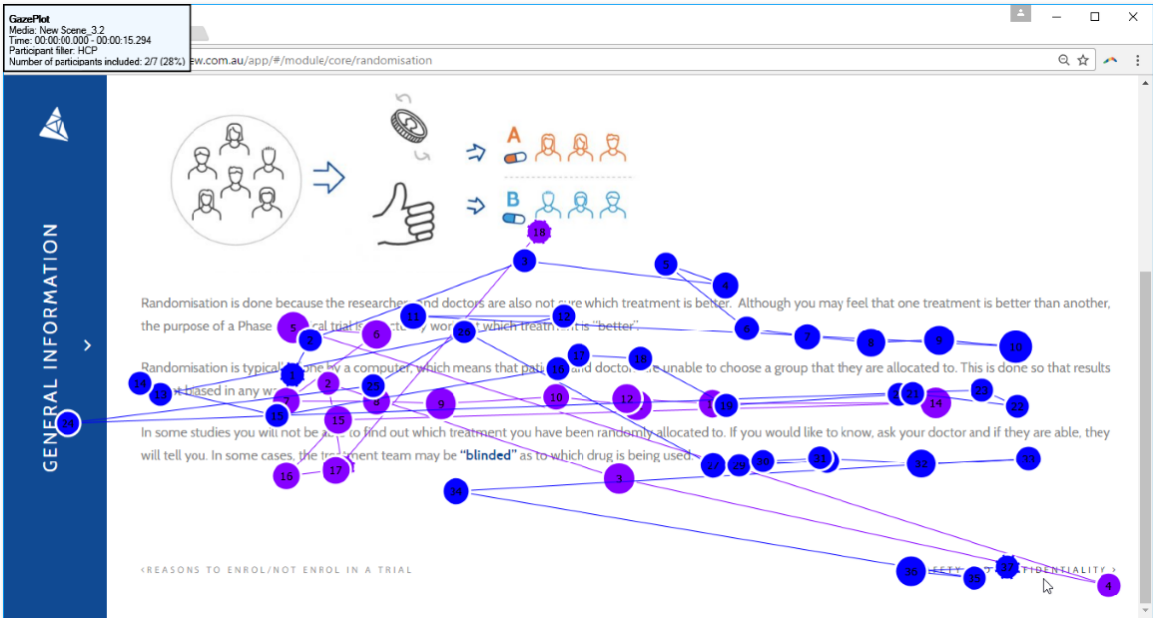
Gaze plot of 2 participants. Different colored circles represent each participant. Circles represent the gaze or fixation of the participant, with larger circles indicating longer fixations. Circles are numbered based on the order of fixation. The line between each fixation point represents the saccade or rapid movement between each fixation. Both participants appeared to be able to easily see the “next” button to navigate to more content. Only 2 participants were able to be used in this gaze plot as Delta is a scrolling website, and other participants did not stop their webpage in the exact same location.

Most participants reported at least some difficulty in navigating the website (3/6 parents; 4/7 parents). Problems included finding the home page and accessing more content. Eye-tracking data suggested that once participants were more familiar with the website, they were easily able to navigate to the next page of content (see Figure 4). In think-aloud interviews, many participants felt that they would have found navigation easier if font and icons were of a larger size and darker color (2/6 HCPs; 6/7 parents). Participants found the values clarification exercise useful, and the participants’ suggested leaning reflected how they felt they were leaning with regards to whether or not to enroll their child in the clinical trial (4/5 HCPs; 6/7 parents):

I think the tool is a helpful way to make a decision. It shows you from the way you’ve answered the questions that you are tending, even if you feel undecided. It gives you options to further think about “Why am I tending that way?,” “What don’t I actually like about the trial?,” and “What more information do I need?"

Mother of a 10-year-old boy with neuroblastoma

After this tool, we would really start talking with each other. The exercise is great—it becomes a tool at that point for more people to really engage.

Mother of a 13-year-old boy with osteosarcoma

**Figure 5:**
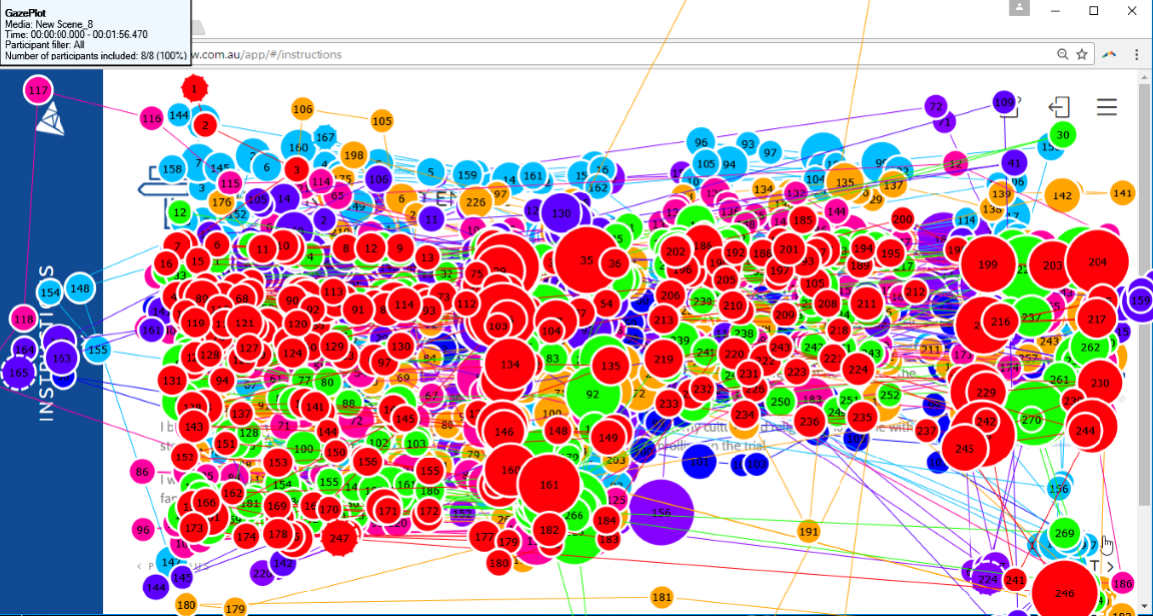
Gaze plot of 8 participants. Different colored circles represent each participant. Circles represent the gaze or fixation of the participant, with larger circles indicating longer fixations. Only 3 participants gazed at the instructions panel on the far left, for a minimal fixation length. Only 8 participants were able to be used in this gaze plot as Delta is a scrolling website, and other participants did not stop their web page in the exact same location.

Eye-tracking analyses showed long fixations for the first few values clarification exercise items and scale. Less time was spent on the last two items of the exercise, suggesting cognitive fatigue. In think-aloud interviews, participants reported feeling unsure of how to use the values clarification exercise (2/5 HCPs; 2/7 parents). Only 5 participants (all HCPs) accessed instructions on how to complete the exercise, with minimal gaze focusing on the instructions panel (see Figure 5). However, most participants (4/6 HCPs; 6/7 parents) found that the exercise was useful and accurate in the direction they felt they were leaning:

I was really impressed—I wasn’t expecting that. It was great. The outcome of enrolling in the clinical trial was how I was feeling. It’s still interesting to see that sentence come up and say that that though

Mother of a 9-year-old girl with acute lymphoblastic leukemia

Participants felt that the user experience of the values clarification exercise could have been improved by providing a clearer introduction and purpose of the exercise (2/6 HCPs; 4/7 parents) and providing more visible instructions (1/6 HCPs; 7/7 parents). See Table 4 for an overview of user testing findings.

### Implementation of User Testing Findings

User testing revealed that both HCPs and parents found Delta to be acceptable and useful. On the basis of the results of the user testing, the lead team reviewed the changes suggested and implemented changes based on consensus. Main changes included replacement of the “home” icon and darker and larger font. See Table 5 for summary of main modifications made. Although some minor issues were raised, overall findings were positive.

**Table 4:** Number of health care professionals (HCPs) and parent who used each Delta function.

Delta functions		Overall (N=13)	HCPs (N=6)	Parent (N=7)
**Values clarification exercise**				
	Completed values clarification exercise	12	5	7
	Completed task without being prompted	5	4	1
	Found exercise was accurate and useful	10	4	6
	Viewed instructions for exercise	5	5	0
**Accessing content**				
	Side panel	6	3	3
	Next button	3	1	2
	Combination of side panel and next button	2	2	0
	Home page	1	0	1
**Acceptability and usability**				
	Reported high acceptability	12	5	7
	Reported high usability	8	4	4
	At least some minor navigational issues	7	3	4
	Suggestion for larger and darker font	8	2	6
**Content**				
	Content easy to read	11	5	6
	Minor word changes suggested	13	6	7
	Read summary boxes first	4	2	2
	Used notes function	0	0	0
	Used glossary function	7	3	4
	Used text-to-speech function	0	0	0
	Skipped at least one page^a^	11	5	6

^a^Data missing from 1 parent.

**Table:** Main modifications based on eye-tracking analyses and think-aloud interviews.

Area^a^	Modification
Content	Minor wording changes made throughout website
Content	Reduction of overlap of content throughout
Content	Clear introduction of purpose of values clarification exercise
Design	Home page more obvious to navigate
Design	Darker color and large-sized font
Design	Values clarification exercise instructions more obvious

^a^Modifications were related to either content or design.

Some of the features within the website were not used (eg, notes page) or were used by approximately half of the sample. We expected this level of use, given that approximately half of the features and functions are used in a typical website [13].

User testing findings should be considered in light of several limitations. We were only able to recruit mothers for this study. However, this may be appropriate feedback, given that mothers tend to take on a greater decisional role than fathers in pediatric oncology [45]. Our findings may also be biased because of our sample having more interest in participating in such research. Finally, as Delta involves pages that scroll, we cannot guarantee the precise accuracy of the eye-tracking analyses in regard to the gaze plots and heat map outputs. Although feedback regarding Delta was positive, we cannot assure ecological validity in that parents would actively engage with the content outside of the user testing given the potential of the Hawthorne effect.

## Discussion

### Study Overview

This paper details the development of Delta, a Web-based decision aid to support families to decide whether or not to enroll in a pediatric oncology clinical trial. We developed Delta using the Agile approach, which included a requirements analysis, planning, design, development, and user testing. We developed Delta iteratively, focusing on short sprints of work. Delta is the first a Web-based decision aid, to the authors’ knowledge, developed using the Agile approach.

### Benefit of Using Agile in Developing a Web-Based Psychosocial Tool

The benefit of using Agile in the development of Delta has allowed for a high level of collaboration between the lead development team, HCPs and researchers, and consumers. In research projects, such as Delta, collaborators are often chosen based on their individual expertise. This aligns well with Agile, as the success of Agile projects is largely dependent on the expertise of the team involved [46].

Involving consumers throughout development and in user testing has been especially important for Delta. The World Health Organization Declaration of Alma Ata states that “people have the right and duty to participate individually and collectively in the planning and implementation of their health care” [47]. When developing patient tools, especially for vulnerable populations as the case for Delta, patients’ and families’ roles should play an active role [48]. The Agile process caters for consumer involvement. Involving patients and families in the development of patient information material may result in more appropriate, sensitive, readable, and understandable information [49]. For Delta and similar research projects, this means that the limited funds available are available to be spent on what is considered most important for families and clinicians.

The benefit of using Agile in the development of Delta has also been in working in short sprints and iteratively. This means that smaller aspects of the website were presented to the developmental team, followed by iterative refinement. Short sprints mean that required changes were usually minor and thus more financially manageable. For Delta, minor issues such as reorganization of content were able to be resolved early into development. Breaking Delta into more manageable units also meant that we were able to focus on detailed yet high-quality development. Quick releases are a major benefit of using Agile [50]. Being able to deliver short sprints of work has encouraged collaborators to become more engaged, provide quality feedback, and ensure deadlines are being met.

### Barriers to Using Agile in Developing a Psychosocial Tool

One of the major limitations of using Agile to develop Delta is because of the slow-moving nature of research. This has limited the ability for short and fast sprints of work. To ensure high quality of product, involvement of clinicians and consumers has been imperative. We constantly adapted our time frames to work within the constraints of the experts involved. The Delta steering committee, however, found difficulties in maintaining constant face-to-face meetings. Agile may be difficult when working with larger teams, especially with more than 20 to 30 members [50]. Even with a team of 13 members such as with Delta, issues arose in regard to meeting deadlines for feedback and having too many meetings. To overcome the difficulty in obtaining feedback at each iteration, we allocated certain tasks to team members based on their expertise and interests. The very hierarchical approach that Agile takes to development may also not work within some workplace cultures [46]. Organizations that have greater bureaucracy and formality may also experience difficulty in fast sprints of work.

With multiple iterations, the project scope is subject to change. Creating accurate budgets and schedules at the start of the project is difficult. For Delta, over the course of development, the scope of the website has almost doubled, from originally being a purely parent website to now including both parent and adolescent versions. Although Agile has allowed for scope creep in a more controlled and manageable manner, the change in scope has been costly and needed to be better budgeted. Some functions that were implemented during early cycles of development can also become redundant as requirements and scope change. The redesign and recoding can add significant costs.

For Delta, we were required to obtain ethics approval to conduct user testing, which pushed back the schedule by several weeks. Difficulties with recruitment, such as booking in times for families to complete the testing at the designated location, also delayed user testing. When working with patients and families, researchers need to be aware of the time to recruit and to conduct user testing.

Although discussions with experts and the literature have guided the development of Delta, sometimes this does not always turn out to be what is logistically possible or what families want. Agile focuses on consumers’ and development teams’ preferences, which meant there was an emphasis on the functional requirements of Delta (eg, text-to-speech function, glossary, and wording). The Research-Based Web Design and Usability Guidelines also encouraged us to consider nonfunctional requirements. Nonfunctional requirements include horizontal (ie, ability to build out and be produced in a variety of capabilities, such as Delta on a computer and also mobile phone compatible) and vertical scalability (ie, increasing resources or capabilities on a single component, such as Delta), security, maintainability, and longevity of Delta. However, when working with Agile, researchers should balance consumer preferences with usability and performance.

### Future Directions

We recommend that researchers can develop a Web-based decision aid using Agile. We suggest that researchers set deadlines for iterations of sprints of work even in the case that the planned work may not be completely finished [51]. We strongly recommend that the 10th Agile principle of “simplicity” (ie, getting “just” enough done as needed for right now) be incorporated. We suggest that if only minimal feedback is provided by a set deadline, development continues as per these deadlines. Given the increase in value after a certain number of iterations reaches saturation [13], this may ultimately reduce the “time-to-market” without impacting quality of work. However, it must be noted that although these deadlines are technically “set,” they do require constant updating in regard to priorities and expected time for completion as the project progresses. We suggest having a *product backlog* of what has been completed and what needs to be done. The product backlog should acknowledge the value (ie, how useful or important is this as part of the project?) and the size (ie, how difficult or long development is expected to take?) of developing the feature or function. Better understanding the value and size can allow researchers to predict the return on investment and potentially reduce costs where possible. The *product backlog* should also note the velocity (ie, the rate of progress or time to complete an iteration) to ensure progress continues at an appropriate pace. We have included a suggested template for reporting the product backlog (see Multimedia Appendix 3). We also suggest future researchers’ budget for multiple iterations throughout development, both in regard to time and cost.

### Conclusions

This paper provides an overview of the development and early testing of Delta, a patient Web-based decision aid for pediatric oncology clinical trial enrollment. We developed Delta using the gold standards of patient decision aids and Web development. In development of decision aids specifically, there is a definite need for guidelines or examples of development processes [52]. This paper has begun to fill this gap in the literature, providing more guidance for researchers looking to develop a Web-based decision aid. Delta acts as proof of concept of the use of Agile for the development of a Web-based decision aid. Aspects of Agile development may be useful to incorporate to ensure the development of a high-quality and high-value project within the constraints of cost, schedule, and scope. The development process detailed in this paper provides a suggested template from which future tools can be developed.

## Appendix

Multimedia Appendix 1 Table of Agile principles and translation to the development of Delta.

Multimedia Appendix 2 Video providing an overview of the Delta website and user-testing.

Multimedia Appendix 3 Table providing recommended backlog template for future researchers.

## Abbreviations

HCP: health care professional
IPDAS: International Patient Decision Aid Standards
