# Supplementary material for: The Development of Delta: Using Agile to Develop a Decision Aid for Pediatric Oncology Clinical Trial Enrollment
Source: JMIR Res Protoc. 2018 May 4;7(5):e119. doi: 10.2196/resprot.9258 (PMC5960036; doi:10.2196/resprot.9258)
Supplement: Multimedia Appendix 1 [file resprot_v7i5e119_app1.pdf]

Supplementary file 1. Translation of Agile Software Development Principle for the development of Delta

	Agile Software Development Principle	Delta response
1	Our highest priority is to satisfy the customer through early and continuous delivery of valuable software.	2-4 week sprints of work
2	Welcome changing requirements, even late in development. Agile processes harness change for the customer's competitive advantage.	Request feedback throughout each stage of process, and avoid delays when changes are suggested
3	Deliver working software frequently, from a couple of weeks to a couple of months, with a preference to the shorter timescale.	2-4 week sprints of work
4	Business people and developers must work together daily throughout the project.	*Outline frequency of required meetings for steering committee in Terms of Reference Agreement
5	Build projects around motivated individuals. Give them the environment and support they need, and trust them to get the job done.	Invited experts in the field with an interest in the project to be on the steering committee
6	The most efficient and effective method of conveying information to and within a development team is face-to-face conversation.	Lead team member meeting held fortnightly and face-to-face
7	Working software is the primary measure of progress.	Tracking of working software during fortnightly lead team member meetings
8	Agile processes promote sustainable development. The sponsors, developers, and users should be able to maintain a constant pace indefinitely.	Estimated time frames of working software, which were constantly updated during fortnightly lead team member meetings
9	Continuous attention to technical excellence and good design enhances agility.	Incorporated both preferences and needs of consumers, with usability and performance requirements (acknowledging the Research-Based Web Design and Usability Guidelines and International Patient Decision Aid Standards)
10	Simplicity--the art of maximizing the amount of work not done--is essential.	*Set deadlines but continue development process even if not all feedback is received
11	The best architectures, requirements, and designs emerge from self-organizing teams.	Organised team in to lead team members and larger steering committee. Allocated tasks based on expertise.
12	At regular intervals, the team reflects on how to become more effective, then tunes and adjusts its behaviour accordingly.	Discussed barriers and facilitators in meeting deadlines during fortnightly lead team member meetings

*Within the research environment, Principle 4 and 10 were difficult to adhere to. Recommendations for future research in addressing these principle are outlined instead
