# Supplementary material for: The Development of Delta: Using Agile to Develop a Decision Aid for Pediatric Oncology Clinical Trial Enrollment
Source: JMIR Res Protoc. 2018 May 4;7(5):e119. doi: 10.2196/resprot.9258 (PMC5960036; doi:10.2196/resprot.9258)
Supplement: Multimedia Appendix 3 [file resprot_v7i5e119_app3.pdf]

Appendix 3. Template for product back-log

**Feature/function**	**Value**[a]	**Size**[b]	**ROI**[c]	**Expected velocity**	**Actual velocity**
e.g. Text-to-speech function	*2*	*1*	*2*	*7 days*	*5 days*

[a]*Value to be rated on a scale of 1-5, with 1=less value or importance and 5=high value or importance.*
[b]*Size to be rated on a scale of 1-5, with 1=simple task or quick to complete 5= difficult task or long to complete*
[c]*Expected return of investment (ROI)=Value/Size (where <0.4 is a questionable ROI and may need to be further considered)*
[d]*Expected velocity=expected time to complete task*
[e]*Actual velocity=actual time to complete task*
